# The Role of Atypical Chemokine Receptors in Neuroinflammation and Neurodegenerative Disorders

**DOI:** 10.3390/ijms242216493

**Published:** 2023-11-18

**Authors:** Hunter G. Lindsay, Colby J. Hendrix, Josue D. Gonzalez Murcia, Christopher Haynie, K. Scott Weber

**Affiliations:** 1 Department of Microbiology and Molecular Biology, Brigham Young University, Provo, UT 84602, USA; 2 Department of Neurobiology, University of Utah, Salt Lake City, UT 84112, USA

**Keywords:** atypical chemokine receptors, cytokines, chemokines, neuroinflammation, neurodegenerative disorders

## Abstract

Neuroinflammation is associated with several neurodegenerative disorders, including Alzheimer’s disease (AD), Parkinson’s disease (PD), and multiple sclerosis (MS). Neuroinflammation provides protection in acute situations but results in significant damage to the nervous system if chronic. Overexpression of chemokines within the brain results in the recruitment and activation of glial and peripheral immune cells which can propagate a cascading inflammatory response, resulting in neurodegeneration and the onset of neurodegenerative disorders. Recent work has identified the role of atypical chemokine receptors (ACKRs) in neurodegenerative conditions. ACKRs are seven-transmembrane domain receptors that do not follow canonical G protein signaling, but regulate inflammatory responses by modulating chemokine abundance, location, and availability. This review summarizes what is known about the four ACKRs and three putative ACKRs within the brain, highlighting their known expression and discussing the current understanding of each ACKR in the context of neurodegeneration. The ability of ACKRs to alter levels of chemokines makes them an appealing therapeutic target for neurodegenerative conditions. However, further work is necessary to understand the expression of several ACKRs within the neuroimmune system and the effectiveness of targeted drug therapies in the prevention and treatment of neurodegenerative conditions.

## 1. Introduction

### 1.1. ACKRs and cCKRs

Our cells utilize a complex network of signaling molecules and receptors to communicate with each other. Among these signaling molecules are cytokines, which predominantly affect cells and tissues within the immune system and influence immune cell activation, differentiation, and migration [1]. A subgroup of cytokines are chemokines, which are small proteins (8–12 KDa) primarily responsible for chemotaxis, or cell migration. Organized based on their N-terminal amino acid sequence, chemokines are categorized into four subfamilies: CC, CXC, CX3C, and XC [2]. Chemokines are produced and secreted by a variety of cells, including immune cells, endothelial cells, and epithelial cells [3]. To bind to these secreted chemokines, cells use surface-expressed conventional chemokine receptors (cCKR), which activate various cell processes, including cell migration, survival, proliferation, and other cytokine release. To activate these processes, cCKRs use both G-protein coupling and β-arrestin activation, resulting in chemokine signaling redundancy [4]. Currently, 23 cCKRs have been identified, grouped according to the chemokines they bind. Aside from cCKRs, atypical chemokine receptors (ACKR) also bind to chemokines and are expressed on a variety of cells. ACKRs and cCKRs are structurally homologous and often bind to similar chemokines. However, the distinction between ACKRs and cCKRs lies in the inability of ACKRs to activate G proteins. As a result, ACKRs lack the downstream signaling necessary for chemotaxis [5,6]. This indifference to G proteins was originally thought to be caused by ACKRs lack of the DRYLAIV motif, a canonical amino acid sequence conserved in other G protein-coupled receptors. However, modifications to the DRYLAIV motif in cCKRs known to activate via G-protein coupling, such as CXCR6, did not disrupt G-protein activation [7]. This suggests modifications to the DRYLAIV motif are not the only determinate in the lacking activation. In addition, restitution of the motif within ACKRs did not fully restore signaling, challenging the original dogma, but data for this are conflicting [8].

### 1.2. Inflammation and Neuroinflammation

ACKRs are expressed on many cell types in various bodily systems [9,10,11,12,13,14,15,16]. Their expression within the immune system has received notable attention with research focusing on ACKRs in the context of cancer, autoimmunity, and infection [17,18,19]. ACKRs possess a unique ability to alter the immune response by modulating the bioavailability and gradients of chemokines. Reliant upon these gradients are adaptive immune cells like B cells and T cells, and innate immune cells like macrophages, neutrophils, natural killer cells, dendritic cells, and monocytes, collectively referred to as leukocytes [20]. Using cCKRs, leukocytes recognize chemokine gradients and migrate through the body to sites of infection or tissue damage. In the event that a pathogen evades our primary immune defenses (an impermeable skin barrier and selectively permeable mucous membranes), cytokines are secreted from the infection site and activate leukocytes which, alongside pathogen virulence factors, contribute to acute inflammation. This acute inflammation is generally associated with the localization of activated immune cells to the site of infection and removal of the pathogens over a short period of time. Occasionally, this inflammation can become chronic because of either repeated trauma, resistant pathogens, or erroneous leukocyte activation and recruitment. Chronic inflammation can result in off-target effects, leading to excessive tissue damage and fibrosis [21].

The immune system and the nervous system are intricately connected via bidirectional pathways. This relationship involves interaction and communication between various cell types, of which an example is mast cells and microglia [22]. Neuropeptides, produced by microglia, can activate mast cells, and worsen mast cell-related diseases such as allergies [23]. In turn, when mast cells respond to immune stress, they can activate microglia which can lead to an inflammatory response in the central nervous system (CNS) [24].

Inflammation within the brain and spinal cord (collectively known as the CNS) is also referred to as neuroinflammation and can be especially dangerous. In the event of tissue damage or infection within the CNS, a combination of neuroimmune cells, cytokines, chemokines, and peripheral immune cells are employed to resolve the issue. The neuroimmune system is primarily composed of microglia, oligodendrocyte, and astrocytes [25]. Microglia are commonly thought of as the resident CNS macrophages and differentiate from hematopoietic common myeloid progenitor cells [26]. A simplified spectrum of microglial polarization contains M1 and M2 phenotypes. M1 microglia are pro-inflammatory, secreting inflammatory cytokines like interleukin (IL)-6 and tumor necrosis factor alpha (TNF-α) [27]. M2 microglia are anti-inflammatory, producing anti-inflammatory cytokines such as IL-10. M2 microglia phagocytose debris, cleaning up the CNS and promoting tissue repair [27]. Once activated, microglia become mobile which allows them to reach the site of injury [28]. These cells have been shown to remain at the site of infection/injury for a long time, releasing cytokines and neurotoxic agents which can cause CNS damage [29]. Although originally presumed to be separate from the neuroimmune system, oligodendrocytes have several strong immune functions. Oligodendrocytes express a wide variety of innate immune receptors and produce and respond to cytokines and chemokines [30]. Other connections to the neuroimmune system are unknown. The final neuroimmune cell type outlined here is astrocytes. These cells maintain the CNS homeostasis via clearance of cell debris, assisting neurotransmitter production, and up-keep of the blood–brain barrier (BBB) [31,32,33]. The BBB is composed of endothelial cells and is a selectively permeable membrane that strictly controls the passage of ions, molecules, and cells between the periphery and the CNS [34]. Like the mucous membrane of the peripheral immune system, an important goal of the BBB is to prevent entry of pathogens and toxins. In cases of CNS infection or damage, cytokines, such as IL-17 and TNF-α, and chemokines are used to increase BBB permeability allowing peripheral immune cell entry [35]. In response to these chemokines, peripheral immune cells will migrate to the BBB to assist with pathogen clearance or tissue repair [36]. While this is beneficial in acute neuroinflammation, infiltrating immune cells can exacerbate chronic inflammation, causing increased neuronal death and contributing to neurological disease progression [37,38,39]. In addition, damage to the BBB via trauma [40,41,42] and oxidative stress [43] enables leukocytes to enter the parenchyma and can exacerbate neuroinflammation.

### 1.3. Neurodegeneration

Neurodegeneration is a culmination of multiple factors and events that result in disrupted CNS homeostasis, cognitive impairment, and neuronal death [44]. Neuronal cell death within the CNS can be caused by mitochondrial malfunction, inflammatory cytokines, and protein dysfunction and can have devastating consequences in neurodegeneration [45]. Age, genetic predisposition, physical trauma, and inflammation all contribute to the progression of neurodegeneration [46,47,48]. Neurological diseases classified as neurodegenerative include but are not limited to Alzheimer’s disease (AD), Parkinson’s disease (PD), multiple sclerosis (MS), Huntington’s disease, and amyotrophic lateral sclerosis [49,50,51]. The prevalence of such diseases is rapidly increasing [52]. Unfortunately, because of the complexity and sensitivity of the CNS, research investigating each neurodegenerative disease is difficult and the exact cause for most has remained elusive. The current accepted view of the cause of AD is that a build-up of extracellular amyloid-β results in amyloid plaques and intraneuronal tau tangles. These plaques and tangles are neurotoxic leading to neuronal cell death and neurodegeneration [53]. However, there has been mounting evidence that amyloid-β plaques do not explain the entirety of AD, opening the doors for further hypotheses [54].

Genomic analysis has identified several AD risk genes that are associated with innate immune functions and increased inflammatory markers; taken together, these data suggest a role of neuroinflammation in AD. The neuroinflammatory hypothesis identifies microglia and astrocytes as the predominate perpetrators, which if defective, impair amyloid-β clearance. They are believed to induce chronic neuroinflammation and neuronal death [55]. The pathogenesis of PD remains elusive. However, theories have pointed to oxidative stress, genetic defects, neurotoxins, neuroinflammation, and metabolic disorders [56,57,58,59,60]. It is likely that a combination of these factors is responsible for PD. Disease progression occurs slowly over many years before detection and diagnosis can prove difficult [61]. Characteristic of PD is Lewy body deposits, an aggregation of cellular protein, at sites of neuron loss [62]. Neurodegeneration within PD damages dopaminergic neurons, resulting in a lack of dopamine production and signaling which causes impaired movement.

MS is perhaps the most characterized of the neurodegenerative diseases discussed here. MS is an autoimmune neurodegenerative disease designated by destruction of the myelin sheath along the neuronal axons by autoreactive immune cells [63]. While the trigger for the autoreactive cell activation is unknown, the pathogenesis that follows is well understood. The destruction of the myelin sheath causes the neuronal axons to degrade, disrupting CNS function [64]. Theories as to the initial trigger of autoimmunity in MS include genetics, geographical location, vitamin D deficiency, age, and viral infection [65,66,67]. A common animal model for MS is experimental autoimmune encephalomyelitis (EAE). In an EAE model, myelin basic protein is injected into a mouse, which induces an autoimmune response in CD4+ T-cells; these cells begin to attack the myelin sheath surrounding motor neurons. Several studies discussed in this review utilize the EAE model. Based on current understanding, neuroinflammation is implicated in AD, PD, and MS, among other neurodegenerative disease.

Given the mounting evidence of neuroinflammation as a contributor to neurodegenerative diseases, the management of neuroinflammation has garnered significant research interest. If a therapeutic agent could restore CNS homeostasis, it could be a potent treatment for neurodegenerative diseases including AD, PD, and MS. Consideration of the ability of ACKRs to modulate extracellular chemokine levels has led them to receive research interest in this pursuit of restoring homeostasis. Therefore, this review discusses the current body of research connecting ACKRs to the modulation of neurodegenerative diseases. Four ACKRs (ACRK1–4) and three putative ACKRs (CCRL2, GPR182, and PITPNM3) have been identified throughout the body. However, only ACKR1–3, CCRL2, and PITPNM3 have cellular expression within the CNS. Therefore, this review does not discuss ACKR4 and GPR182. In addition, no research has connected PITPNM3 to neurodegeneration and therefore it too is excluded from the current review. This review begins by discussing the current known functions of ACKRs. Each ACKR (ACKR1–3 and CCRL2) will then be summarized individually, focusing on their cellular expression within the CNS, their unique molecular mechanisms, and their known and putative binding partners (summarized in Supplementary Materials Figure S1). We then summarize the current body of research concerning the role of each ACKR as it relates to neurodegeneration. Finally, we discuss areas of future research and potential connections to explore. Based on the evidence gathered here, ACKRs play a unique role in maintaining the equilibrium of neuroinflammation and serve as an appealing drug target for therapies for neurodegeneration. More research is ultimately needed to understand ACKRs full role in managing neuroinflammation in neurodegenerative diseases.

## 2. ACKR Functions

**Figure 1:**
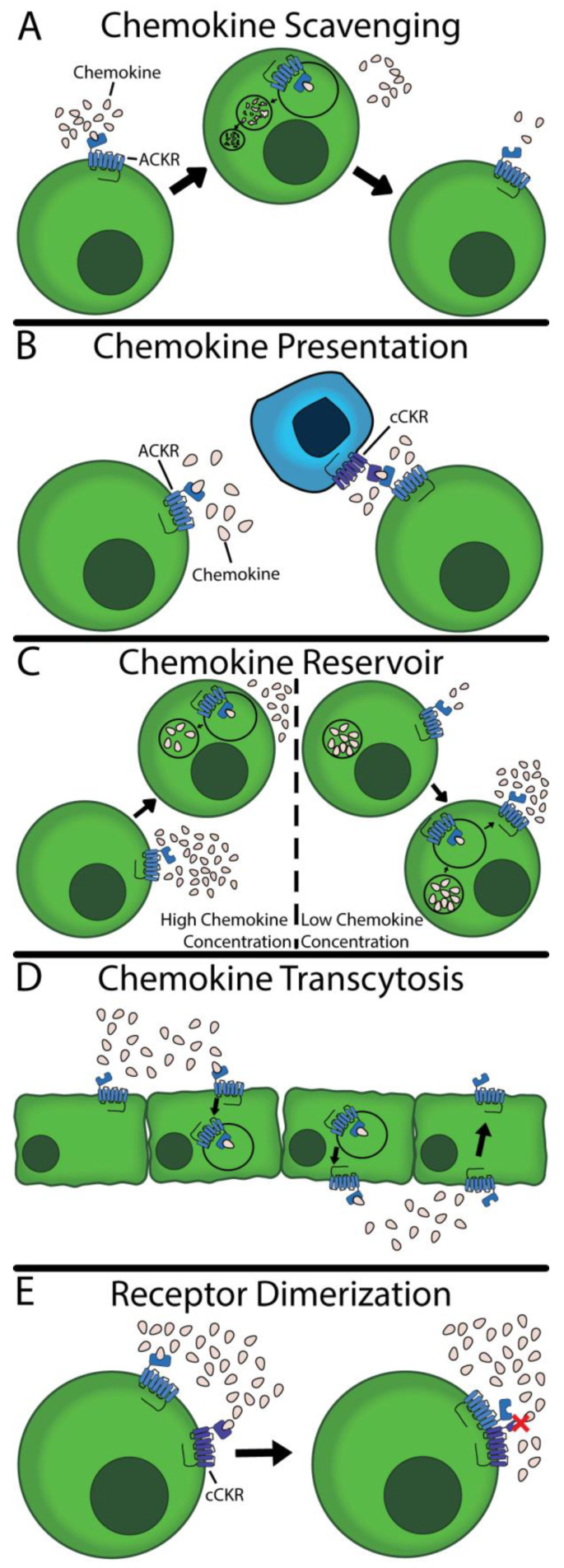
Five functions of ACKRs. (**A**) ACKRs can bind, internalize, and degrade their respective ligands. The ACKRs are subsequently returned to the surface to repeat the process. (**B**) ACKRs can remain fixed on the surface, holding and presenting chemokines to other cells. These presented chemokines can bind to cCKRs and initiate downstream pathways. (**C**) Cells with ACKRs can have a vacuole with stored chemokines. Using this, ACKRs can buffer chemokine concentration, removing or releasing chemokines to maintain homeostasis. (**D**) ACKRs can facilitate the transcellular migration of chemokines. They will bind on the basolateral side of a membrane and transport the chemokines to the apical side. (**E**) ACKRs can dimerize with other receptors to inhibit signaling or ligand binding. The red X indicates an inhibition of cCKR activity.

While ACKRs are unable to facilitate chemokine-directed cell migration or trigger G protein-mediated signaling in the traditional sense, they nonetheless play an important role regulating chemokines. Most significantly, ACKRs are widely documented to manage local chemokine concentrations, thereby impacting cell migration and inflammatory processes. Five ACKR functions have been documented throughout the body to fulfill this modulatory role, which are discussed in detail below. They are described in Table 1 and are illustrated in Figure 1. For unknown reasons, any given ACKR has only been documented to perform a handful of these functions.

### 2.1. Chemokine Scavenging

The most widely cited function of ACKRs is that of chemokine scavenging (Figure 1A) [78]. In this specific function, the ACKR binds to its respective ligand; the complex is then internalized and transported to the lysosome, where the ligand is degraded. This process may be β-arrestin dependent [71,79], although recent work has shown that β-arrestin is not entirely necessary [74,80,81,82]. In fact, Montpas et al. demonstrated that a point mutation in ACKR3 slowed scavenging in both the presence and absence of β-arrestin [82]; taken together, these data suggest the optionality of β-arrestin and suggest the existence of an unidentified effector. Following chemokine degradation, the ACKR returns to the surface to scavenge more chemokines [8,71]. This unique property enables a low expression of ACKRs to effectively scavenge large quantities of chemokines, resulting in lower concentrations of chemokines and impacting other cCKR-mediated processes.

### 2.2. Chemokine Presentation

ACKRs also can remain expressed on the surface of cells without initiating signaling nor internalizing ligands. Instead, ACKRs can act as decoy receptors, presenting and concentrating their ligands (Figure 1B) [83,84]. This process provides several biologically relevant outcomes. First, chemokine presentation facilitates the binding of chemokines to other cell types via direct presentation. Second, by binding, ACKRs create a locally higher chemokine concentration. This concentration can facilitate cCKR binding to their ligand, increasing conventional signaling [83].

### 2.3. Chemokine Reservoir

ACKR1 is unique in its capacity to act as a chemokine reservoir (Figure 1C). Unlike ACKR2–4, ACKR1 internalizes but does not scavenge ligands [10,68]. Rather, the internalized ligands are moved into and stored in caveolae [10]. Using these caveolae, ACKR1 can regulate the extracellular ligand gradient via absorption or release, acting as a chemokine reservoir [11,85,86,87]. This role enables ACKR1 to react to the changing condition by finely tuning extracellular chemokine concentrations. The exact mechanism of the regulation of absorption and release is not known within the CNS.

### 2.4. Chemokine Transcytosis

Like the chemokine reservoir, ACKR1 is the only documented receptor to perform chemokine transcytosis (Figure 1D). In this specific function, the ACKR binds the ligand, and the complex is internalized. Instead of being transported to the lysosome, the complex is targeted into caveolae. The caveolae are routed transcellularly where the caveolae merge with the cell membrane and the ligand is released. The ACKR is returned to the original side of the cell [10]. Of note, this transport has only been documented in the apical to basal direction and the transport has been reported in vascular endothelial cells and the BBB. Pruenster et al. [10,11] subsequently showed this transport facilitates leukocyte trafficking across the endothelial barrier in healthy subjects but it is unknown if a similar role is played at the BBB.

### 2.5. Receptor Dimerization

The final function of ACKRs is the dimerization to cCKRs (Figure 1E). This dimerization results in a loss-of-function of the cCKRs, resulting in downstream effects. ACKR1 has been shown to heterodimerize to CCR5 [69], ACKR3 with CXCR4 [88], and CCRL2 with CXCR2 [89], acting as a functional antagonist. This mechanism provides another powerful method to regulate the immune response, inhibiting certain cCKR-dependant pathways. No work to-date has explored this relationship within the CNS, nor how such a relationship affects neuroinflammatory conditions.

Overall, ACKRs perform several diverse functions and the specific role that each ACKR takes on has significant physiological effects. However, it is not currently known why a given ACKR will perform one function over another at any given time.

## 3. ACKR1 (or DARC)

**Figure 2:**
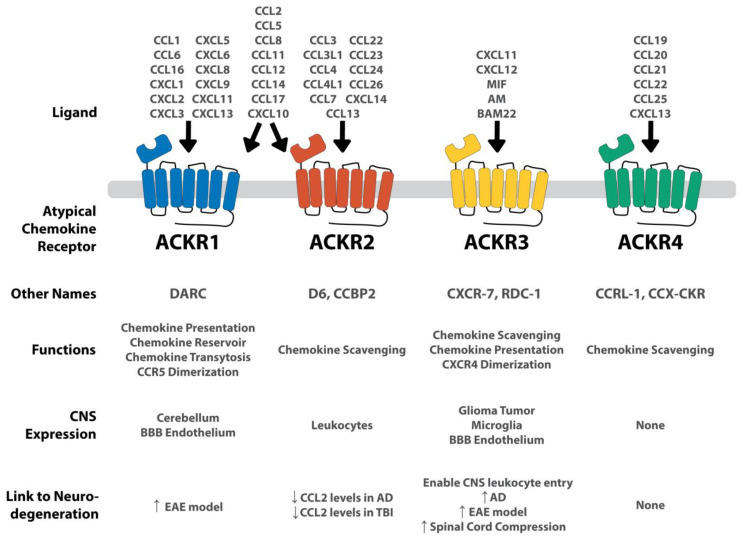
Summary of each ACKR and its connection to the CNS and to neurodegenerative conditions. ACKRs are expressed on a variety of CNS tissues. ACKR1 and ACKR2 are very promiscuous, binding to several different CXCL and CCL chemokines. ACKR3 is more selective, binding to CXCL11 and CXCL12, as well as several non-chemokine peptides including MIF, AM, and BAM22. ACKR4 binds a handful of CCL ligands. Concerning the connection to neurodegeneration, the arrows represent the up-regulation of the ACKR in the given neurologic condition. In ACKR2, the down arrow indicates the decreased levels of significant chemokines given the ACKR’s presence. Both ACKR1 and ACKR3 are upregulated in several neurodegenerative conditions, whereas ACKR2 modulates CCL2 levels. ACKR4 shows no CNS expression and therefore, no apparent connection to neurodegeneration. Adapted from Szpakowska et al. [93].

ACKR1, also known as Duffy antigen receptor for chemokines (DARC), was first discovered in 1950 as a blood antigen group [90], but was later associated with interactions between inflammatory chemokines and erythrocytes (Figure 2) [91]. Subsequent ACKR1^−/−^ mouse studies identified ACKR1 as being important in modulating the immune response to endotoxins and chronic inflammation [85,92]. To accomplish this immune modulation, ACKR1 performs several of the ACKR functions seen in Figure 1 and Table 1. It acts as a chemokine presenter, facilitates chemokine transcytosis, acts as a chemokine reservoir, and dimerizes with CCR5 [10,11,68,69]. These diverse functions enable ACKR1 to play a unique role in inflammation management throughout the body.

Concerning cellular expression, ACKR1 is mostly found on endothelial tissues, such as in postcapillary venules in the kidney and in the spleen [10,12]. Within the CNS, ACKR1 is expressed on endothelial cells in the BBB [11,12].

### 3.1. ACKR1 Binding Partners

ACKR1 functionally binds to CCL1, 2, 5, 6, 8, 11, 12, 14, 16, 17, and CXCL1, 2, 3, 5, 6, 8, 9, 10, 11, and 13 [94]. Most of these chemokines are pro-inflammatory, aiding in neutrophil and monocyte trafficking and activation.

### 3.2. ACKR1 and Neurodegeneration

The connection between ACKR1 and neurodegeneration has remained almost entirely unexplored. Minten et al. have the only documented research connecting ACKR1 with MS using an EAE mouse model. The results of Minten et al. showed ACKR1 experienced increased expression in microvascular endothelial cells within the CNS before and during EAE-onset [11]. Other research has documented similar ACKR1 expression and found ACKR1 was involved in the leukocyte recruitment across the BBB via chemokine transport to the luminal side of the brain [10]. Minten et al. suggested a similar method may occur, enabling CNS leukocyte infiltration; to test this, they showed CCL2 and CCL5 were transported to the luminal side in an in vitro model. These chemokines end up in the blood, recruiting leukocytes and enabling their infiltration. ACKR1 also showed increased expression in MS white matter. Finally, ACKR1^−/−^ C57BL/6 mice exhibited significantly ameliorated EAE clinical scores. To elucidate if these ameliorated clinical scores were due to CNS endothelial ACKR1 or erythrocytes which express ACKR1, Minten et al. transplanted C56BL/6 bone marrow into irradiated ACKR1^−/−^ mice and vice versa. The ACKR1^−/−^ mice, who had wild-type erythrocytes, had similarly improved clinical scores as before. These data indicate the improved clinical score is because of the missing ACKR1 on CNS endothelial tissues as opposed to erythrocytes [11]. These results suggest ACKR1 is a pro-inflammatory receptor that facilitates the transport of chemokines to the luminal side of the BBB.

## 4. ACKR2 (or D6)

ACKR2, also known as D6 or chemokine binding protein 2 (CCBP2), was first isolated and cloned from placenta samples in the 1990s (Figure 2) [6,95]. Of the ACKR functions (Figure 1 and Table 1), ACKR2 acts as a chemokine scavenger, targeting a variety of pro-inflammatory chemokines [70,71]. This function makes ACKR2 an appealing target for inhibition and modulation of inflammation. ACKR2 is commonly expressed on lymphatic endothelial cells in the periphery and on leukocytes which enter the CNS. Low levels of ACKR2 expression have been detected on peripheral CD8^+^ T cells and monocytes while high expression has been found on peripheral, naïve CD4^+^ T cells and on antigen presenting cells (APCs) such as dendritic cells and B1 B cells [9,96].

### 4.1. ACKR2 Binding Partners

ACKR2 functionally binds to CCL2, 3, 3L1, 4, 4L1, 5, 7, 8, 11, 12, 13, 14, 17, 22, 23, 24, 26, and CXCL10 and 14 [70,94,97,98,99,100]. Most of these chemokine ligands are pro-inflammatory, although a few have both pro-inflammatory and homeostatic properties.

### 4.2. ACKR2 and Neurodegeneration

A genome-wide association study (GWAS) conducted by Kauwe et al. looked at 59 AD-related CSF analytes in relation to single nucleotide polymorphisms (SNP) [101]. Analyzing 574 patient samples, their results showed an increase of CCL2 levels in patients with a mutated form of ACKR2, resulting from an SNP causing a single amino acid change (V41A) [101]. CCL2 is a pro-inflammatory chemokine implicated in neurodegenerative disease progression [102,103]. Building from this study, Murcia et al. sought to document the specific mechanism responsible for the GWAS findings [104]. They found that ACKR2-V41A has decreased binding to CCL2 as well as CCL4, another pro-inflammatory chemokine implicated in neurodegenerative disease progression [105]. Additionally, in the presence of chemokine, ACKR2-V41A had significantly reduced surface expression whereas unstimulated cells had similar expression levels. Murcia et al. suggested this is due to impaired receptor recycling to the surface. These studies suggest an important anti-inflammatory role of functioning ACKR2 in the resolution of inflammatory chemokines in AD.

Woodcock et al. examined the role of ACKR2 within traumatic brain injury (TBI) [106]. Samples collected postmortem from human TBI victims showed no change in ACKR2 mRNA transcripts as compared to patients who died of unrelated causes. However, in patients who survived for more than 8 h after TBI, a gradual increase in ACKR2 transcripts was observed. A significant positive correlation between ACKR2 expression and survival time was calculated. Transitioning to a TBI mouse model, Woodcock et al. induced TBI in wild type (WT) or ACKR2 knockout mice (ACKR2^−/−^). They reported a significant difference in immediate mortality rate; WT mice had a 25% mortality rate while ACKR2^−/−^ suffered a 46% mortality rate. Additionally, brain lesion volumes in ACKR2^−/−^ mice 1 day after TBI were significantly larger. Interestingly, ACKR2^−/−^ showed no difference in functional recovery over 1 week when compared to its WT counterparts. Also examining ACKR2 within TBI using a rat model, Quan et al. combined modification of ACKR2 expression with sensory integration therapy, a type of occupational therapy, and proposed treatment for TBI [107]. Their study found that induced overexpression of ACKR2 within the CNS in combination with sensory integration therapy improved rat cognitive function after TBI. Rats with ACKR2 upregulation had less edema in the brain and decreased levels of CCL2, IL-1β, and TNF-α. The results from Woodcock et al. and Quan et al. suggest ACKR2 plays a critical role in resolving acute inflammation and aids in recovery from TBI.

Modified ACKR2 expression has also been studied in an EAE model, with Liu et al. using an ACKR2^−/−^ mouse model [108]. Interestingly, ACKR2^−/−^ mice showed lessened disease severity. When immune cells from the lymphoid tissue of an EAE-induced ACKR2^−/−^ mouse were transferred to an ACKR2^+/−^ mouse, disease severity was less than that of ACKR2^+/−^ mice receiving cells transferred from EAE-induced ACKR2^+/+^ mice. Liu et al. also observed aggregation of dendritic cells at the subcutaneous injection site of EAE-inducing peptide. Taken together, Liu et al. concluded that the lessened disease severity in ACKR2^−/−^ mice was due to lessened antigen presentation. This was attributed to impaired migration of antigen presenting cells (APC). ACKR2 is expressed in lymphatic endothelial cells and controls chemokine concentrations to enable proper APC migration to lymphatic tissues [109]. However, a later study by Hansell et al. [70] also examining the impact of ACKR2^−/−^ in EAE models, reported results in stark contrast to those of Liu et al. Hansell et al. reported a slightly increased disease severity in ACKR2^−/−^ mice and observed no difference in DC accumulation at the injection site. Drawing a single conclusion from these studies is complicated. The conflicting results demand more investigation to determine the precise role of ACKR2 in EAE and MS.

The current research of ACKR2 within neuroinflammation prompts a story of inflammatory modulation. Proper management of inflammatory chemokines is key to preventing chronic inflammation within the CNS. ACKR2 poses an attractive target for targeted modulation of neuroinflammation due to its scavenging ability and affinity for a myriad of pro-inflammatory chemokines.

## 5. ACKR3 (or RDC1 or CXCR7)

ACKR3, otherwise known as RDC1 and CXCR7, has one of the most prominent roles of ACKRs within the CNS (Figure 2). The receptor has high-affinity binding to several CXC chemokines as well as other non-chemokine molecules. Of the ACKR functions (Figure 1 and Table 1), ACKR3 acts as a chemokine scavenger, chemokine presenter, and dimerizes with CXCR4 in an antagonistic manner [72,73].

ACKR3 is expressed in smooth muscle of venules and arterioles and in various leukocytes, including T cells, B cells, dendritic cells, and epithelium. Within the CNS, ACKR3 has high expression in glioma tumor cells, microglia, and tumor-associated vascular endothelium [16,110,111]. More specific studies have identified that neurons, astrocytes, oligodendrocyte, and endothelial cells all express ACKR3 [112,113]. Concerning localization, ACKR3 is not isolated to a specific region, but can be found throughout the CNS, including in the cerebral cortex, hippocampus, hypothalamus, cerebellum, and spinal cord [112]. This diverse neuro-expression suggests an essential role of ACKR3 in CNS functioning.

### 5.1. ACKR3’s Binding Partners

ACKR3 has been shown to functionally bind to CXCL11, CXCL12, macrophage migration-inhibitory factor (MIF), adrenomedullin (AM), and bovine adrenal medulla 22 (BAM22) [72,114,115,116,117]. Although ACKR3 shares structural similarities to CXCR2 and CXCR4, ACKR3 binding affinity to CXCL12 is about 10-fold higher than the affinity of CXCR4 [72,118].

### 5.2. ACKR3 and Neurodegeneration

To date, the most significant connection between ACKR3 and neurodegeneration has been identified in modulating leukocyte infiltration into the CNS. Normally, this function plays a neuroprotective role, enabling leukocytes to clear the brain of infection. ACKR3 has been shown to internalize and degrade CXCL12 within the CNS; low levels of CXCL12 result in a permeabilized BBB which results in leukocyte entry [72,117,118]. These leukocytes help clear the stimuli, but unchecked or repeated infiltration can lead to neurodegenerative diseases [119,120]. Hence, ACKR3 plays an essential role as a gatekeeper to the CNS; an upregulation of ACKR3 allows greater leukocyte infiltration and greater neuroinflammation. This role has been implicated in several neurodegenerative diseases.

Several publications have documented the upregulation of ACKR3 on developing astrocytes in the CNS of EAE mice [120,121]. In addition, Cruz-Orengo et al. further document an increased expression of ACKR3 in the CNS microvasculature at the BBB of EAE mice compared to control mice. This increased ACKR3 expression results in lower CXCL12 levels, increased BBB permeability, and increased leukocyte entry [120,122]. The pro-inflammatory connection between ACKR3 and EAE has driven many studies to identify the effect of an ACKR3 antagonist on the progression of EAE. CCX771, one such antagonist, was shown to improve overall disease scores and decrease leukocyte entry into the CNS [120]. In a follow-up study, Cruz-Orengo et al. showed CCX771 enhanced overall recovery regardless of the initial condition of the mouse. This specific recovery was not due to myelin recovery but axon preservation [122]. ACT-1004-1239, another ACKR3 antagonist, showed similar results, decreasing disease clinical scores, and increasing the survival rate; significantly fewer leukocytes infiltrated the CNS. Amazingly, ACT-1004-1239 also encouraged remyelination of the previously damaged neurons, leading to not just an arrested onset but recovery [123].

Other studies have looked at neuroinflammation more generally in connection to ACKR3. High mobility group box 1 (HMGB1) is an endogenous alarmin often associated with neurodegenerative disorders, including AD and PD [124]. Das et al. treated mice with HMGB1 to induce neurodegeneration. Following HMGB1 treatment, ACKR3 silencing RNA (siRNA) was administered to knockdown ACKR3. ACKR3 knockdown was associated with increased levels of pro-inflammatory receptors. ACKR3 agonists appear to inhibit HMGB1-induced immune-cell infiltration, M2-to-M1 microglial conversion, neuronal apoptosis, and lessen memory impairment [16]. Interestingly, these results, which infer that ACKR3 is anti-inflammatory, seem to contradict the findings in EAE and those connecting ACKR3 with BBB permeability, which suggest ACKR3 is pro-inflammatory.

Finally, ACKR3 shows increased expression in the hippocampus of AD patients [121]. Aside from this finding, no further research has been done connecting AD and ACKR3. In all these experiments, the exact functions ACKR3 plays in each of these neurodegenerative conditions is unknown, whether that be as a chemokine sink, another function, or no function. Taken together, the effectiveness of both ACKR3 agonists and antagonists in neurodegenerative diseases highlights the complexities of the CNS. It exists in a delicate equilibrium, where either extreme can result in neuroinflammation and neurodegeneration. Therefore, each neurodegenerative condition must be studied to understand if an agonist or antagonist is needed to return the CNS to equilibrium. ACKR3 is a potent and potentially highly effective drug target in managing chemokine levels within the brain.

## 6. Putative ACKRs

**Figure 3:**
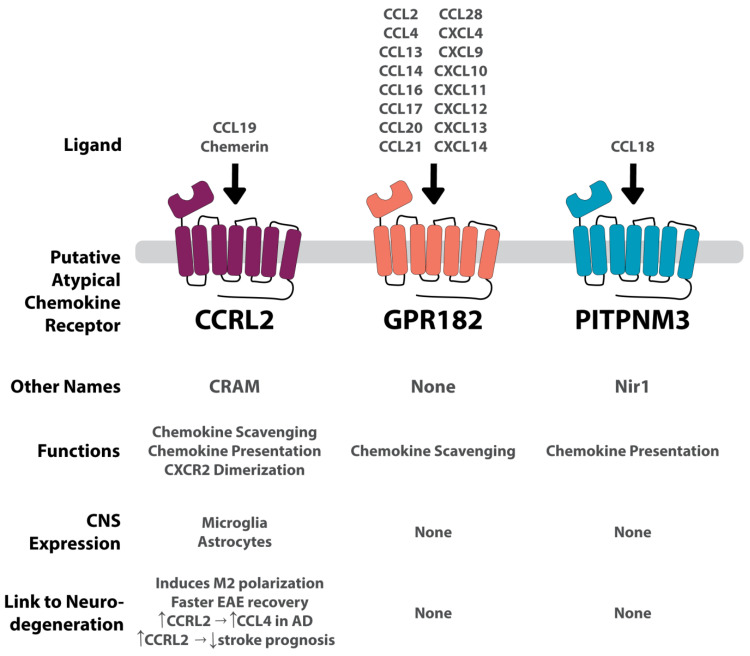
Summary of each putative ACKR and its connection to the CNS and to neurodegenerative conditions. GPR182 is a highly promiscuous receptor binding to several CCL and CXCL chemokines. Conversely, CCRL2 and PITPNM3 are more conservative, binding to one chemokine each. CCRL2 also binds to the non-chemokine, chemerin. GPR182 and PITPNM are not expressed in the CNS, and therefore no apparent connection to neurodegeneration has been found. CCRL2 is expressed on microglia and astrocytes. Its connection to neurodegenerative diseases is convoluted, increasing prognosis in an EAE model, and increasing CCL4 in AD, but also worsening stroke prognosis. Vertical arrows indicate an increase or decrease. Horizontal arrows indicate associative relation.

Aside from the official ACKRs mentioned above, other receptors have been proposed as members of the ACKR family. Like the ACKRs mentioned above, they lack G protein coupling and perform several of the ACKR functions (see Figure 1). However, they each differ slightly from the rest of the ACKR family and are awaiting IUPHAR (International Union of Basic and Clinical Pharmacology) recognition as an ACKR. They are summarized in Figure 3.

## 7. Putative ACRK: CCRL2 (or CRAM)

CCRL2, also known as CRAM, was first discovered in 1998 as an LPS-inducible C-C receptor located on murine macrophages (Figure 3) [15]. Two isoforms for CCRL2 exist, A and B; isoform A (CRAM-A) is less abundant than isoform B (CRAM-B). CRAM-B is a truncated protein with 12 fewer amino acids from the C terminal end verses CRAM-A [83]. CCRL2 is known to preform three ACKR functions (Figure 1 and Table 1): chemokine scavenging, chemokine presentation, and receptor dimerization [75,76,77]. CCRL2 has been shown to dimerize with CXCR2 [89]. To date, it remains a putative ACKR, pending approval.

CCRL2 is expressed in a diversity of cells, including neutrophils, monocytes, macrophages, basophils, mast cells, PMNs, CD4^+^ T cells, pro- and pre- B cells, dendritic cells, NK cells, CD34^+^ progenitor cells, epithelium and endothelium cells [15,76,83,84,125,126,127,128,129,130]. Upon stimulation with pro-inflammatory signals like LPS, CCRL2 has been shown to be upregulated in macrophages and neutrophils [15,76,126]. Within the CNS, CCRL2 is expressed on microglia and astrocytes under inflammatory conditions [128,129].

### 7.1. CCRL2’s Binding Partners

CCRL2’s only definitively shown binding partner is chemerin, which is not a chemokine but is a chemoattractant protein [77,131]. Chemokine like receptor 1 (CMKLR1, also known as ChemR23) and G protein-coupled receptor 1 (GPR1) also share affinity for chemerin. It has been suggested that CCRL2 also possesses affinity for CCL5 and CCL19, though neither have been well established [76,77,131,132].

### 7.2. CCRL2 and Neurodegeneration

A study by Mazzon et al., examining the effect of CCRL2 in the EAE model, found an association between increased levels of CCRL2 during the development of EAE [133]. mRNA transcripts of CCRL2 were increased after EAE induction. CCRL2^−/−^ mice not only had a lengthened recovery but a worsened phenotype with increased mortality. Immunohistochemical staining of the brain 33 days post induction showed CCRL2^−/−^ mice had higher peripheral T cell infiltration as well as increased myelin loss. Mazzon et al. implicated CCRL2 in M1/M2 mononuclear cell polarization as well as T cell activation. M1 markers were higher in the CCRL2^−/−^ mice, while M2 markers emerged much later in CCRL2^−/−^ mice, suggesting a delayed resolution response. Mazzon et al. concluded that CCRL2 may play a role in limiting CMKLR1 expressing cells (i.e., dendritic cells, macrophages, and NK cells) from infiltrating into the CNS. Studies have found impaired CMKLR1 expression in mice in an EAE model showing attenuated disease progression and severity [134,135]. Thus, CCRL2 may suppress neuroinflammation through competition with CMKLR1.

The GWAS conducted by Kauwe et al. discussed previously also reported that a mutated version of CCRL2 (CCRL2-V180M) was associated with decreased CCL4 levels in the CNS [101]. CCL4 is associated with an increased risk for AD [136].

Taken together, the data connecting CCRL2 to neurodegenerative conditions demonstrate a protective role. CCRL2 appears to compete with CMKLR1 for ligand availability, altering the infiltration of peripheral immune cells and subsequent neuroinflammation. In addition, the results presented by Kauwe et al. suggest a potential involvement with pro-inflammatory chemokines.

## 8. Discussion

Our understanding of the role of atypical chemokine receptors in neurodegenerative diseases is limited to relatively few studies. However, the findings to date frame ACKRs as influential players in neuroinflammation and neurodegeneration. Not all ACKRs play the same role, however. ACKR1, ACKR2, and CCRL2 appear to reduce inflammation and attenuate or resolve neurodegeneration in a variety of diseases. ACKR3 currently has conflicting data but seems likely to escalate neurodegenerative symptoms. Importantly, these observations are trends; because of the complexities of neuroinflammation and CNS homeostasis, each ACKR must be studied in the context of each specific neurodegenerative disease to understand its pro- or anti-inflammatory effects.

While some links have been made between ACKRs and neurodegenerative conditions, the exact mechanism informing these links was not addressed in many of the studies discussed. Based on the current body of research, the most significant and likely pathway is as follows: ACKRs modulate CSF chemokine levels. Changes in these chemokine levels impact cCKR binding and signaling which then impacts certain immune processes. Ultimately, this cascade of events impacts the onset of neurodegenerative diseases. Understanding this specific cascade for each ACKR in the individual neurodegenerative disease could reveal novel therapeutic targets. In addition, no study examined above identified the specific ACKR function that facilitated the given phenotype. Understanding these functions may further elucidate the mechanism of action.

In general, ACKRs can efficiently modulate CSF chemokine levels. In addition, the ligands of ACKRs are highly implicated in the prognosis of neurodegenerative conditions. These two observations have been studied independently, but the bridge between them remains elusive; it merits further research to connect ACKRs and their ligands to neurodegenerative conditions. Several potential connections could be imagined and are worth exploring.

## 9. Conclusions

The connection between ACKRs and neurodegenerative diseases remains mostly elusive due to a lack of research and mechanistic insights. There is a much greater understanding of the role of ACKRs in immunology and oncology fields, which has led to proposals for them as possible drug targets for different diseases. All classes of ACKRs have unique interactions between ligands and receptors. These interactions can regulate the immune response and manage cytokine production and expression, cell migration, cell metabolism, and cell homeostasis. In the brain, these roles are critical in maintaining the delicate equilibrium and the resulting normal cognitive functions. However, in cases of neurodegeneration, the immune system has failed to maintain this balance. Given the projected rise of these neurodegenerative diseases over the next several decades, it is increasingly pertinent to discover novel strategies to restore homeostasis in the CNS.

ACKRs, with their immunomodulatory roles, could be an appealing target to return immune balance within the CNS in these neurodegenerative conditions. Specifically, some ACKRs play a unique role as a chemokine reservoir, often removing pro-inflammatory cytokines from the extracellular space. On the other hand, some ACKRs can facilitate chemokine transcytosis, enabling leukocyte infiltration downstream. In both cases, ACKRs play an important role in the fine-tuning of the immune system. Considering these important regulatory functions and the CNS expression of ACKR 1–3 and CCRL2, they appear to have the most potential in modulating neuroinflammation. However, the current body of research has been sparse. Therefore, further research is needed on ACKR1–3 and CCRL2 in connection to neurodegenerative diseases. If connections are found, ACKRs could be an appealing drug target, with their immunomodulatory effects, CNS expression, and potentially fewer side effects. Ultimately, it is necessary to further investigate how ACKRs are possible biomarkers and drug targets to modulate neurodegenerative diseases.

## Figures and Tables

**Table 1:** General functions of ACKRs. ACKR2–4 possess chemokine scavenging ability. ACKR1 and CCL2 can present chemokines. ACKR1 is the only receptor that facilitates chemokine transcytosis and acts as a reservoir. ACKR1, ACKR3, and CCRL2 have all been shown to dimerize with other receptors. GPR182 and PITPNM3 have been excluded due to their lack of connection to the CNS. Sources: ACKR1 [10,11,68,69], ACKR2 [70,71], ACKR3 [72,73], ACKR4 [74], and CCRL2 [75,76,77].

Functions	Which ACKRs	Purpose
Chemokine Scavenging	2, 3, 4	Remove and degrade chemokines from the extracellular space.
Chemokine Presentation	1, CCRL2	Hold chemokines in place for presentation to other cells.
Chemokine Reservoir	1	Act as a chemokine buffer, removing and replacing extracellular chemokines to maintain the appropriate concentration.
Chemokine Transcytosis	1	Facilitate transcellular migration to move chemokines from the abluminal to the luminal side of various membranes.
Receptor Dimerization	1, 3, CCRL2	Dimerize with another receptor to block ligand binding or signaling.

## Supplementary Materials

The following supporting information can be downloaded at: https://www.mdpi.com/article/10.3390/ijms242216493/s1.
