# Supplementary material for: The Role of Atypical Chemokine Receptors in Neuroinflammation and Neurodegenerative Disorders
Source: Int J Mol Sci. 2023 Nov 18;24(22):16493. doi: 10.3390/ijms242216493 (PMC10671188; doi:10.3390/ijms242216493)
Supplement: Supplementary file 1 [file ijms-24-16493-s001.zip › ijms-2701622-supplementary.pdf]

Chemokine-Receptor Binding Interactions

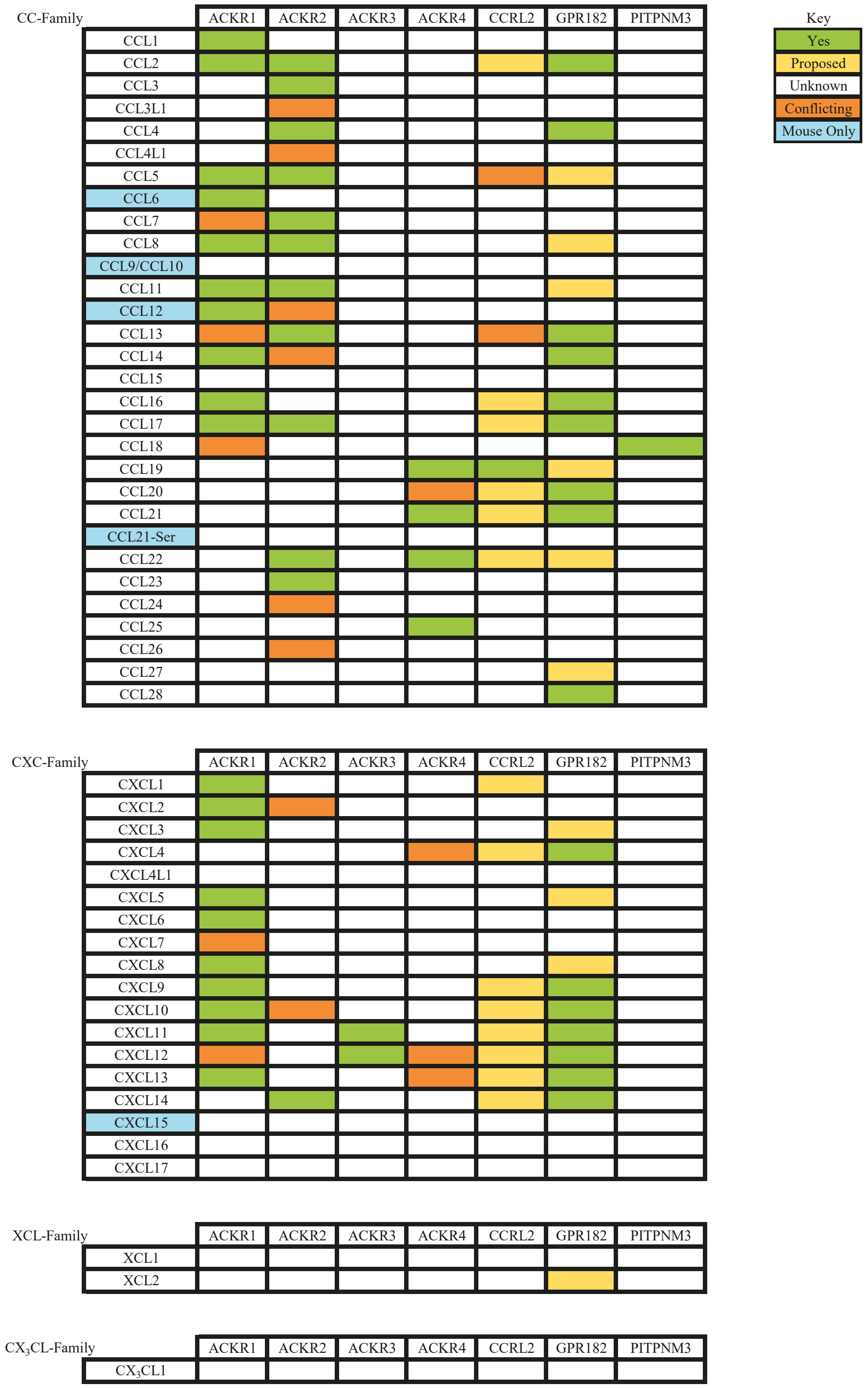

Figure S1. Summary of current known and proposed binding partners for each ACKR. Each confirmed binding is represented in green for the given receptor. Yellow indicates a proposed binding partner as compared to other cCKRs that show similar motifs. Orange indicates conflicting data of binding. White indicates no known information about binding. Blue indicates the binding results have only been shown in mice. ACKR1, ACKR2, and GPR182 are very promiscuous, while the other receptors are more restrictive in their binding associations.
